# Relationship between vascularity, age and survival in non-small-cell lung cancer.

**DOI:** 10.1038/bjc.1997.562

**Published:** 1997

**Authors:** L. M. Chandrachud, N. Pendleton, D. M. Chisholm, M. A. Horan, A. M. Schor

**Affiliations:** Department of Dental Surgery and Periodontology, Dental School, University of Dundee, UK.

## Abstract

**Images:**


British Journal of Cancer (1997) 76(10), 1367–1375
© 1997 Cancer Research Campaign

### Relationship between vascularity, age and survival in non-small-cell lung cancer

**LM Chandrachud[1], N Pendleton[2], DM Chisholm[1], MA Horan[2] and AM Schor[1]**

[1]Department of Dental Surgery and Periodontology, Dental School, University of Dundee, Park Place, Dundee DD1 4HR; [2]Department of Geriatric Medicine, University of Manchester, Hope Hospital, Salford M6 8HD, UK

**Summary** Lung tumours in the elderly show reduced growth potential; impaired angiogenesis may contribute to this phenomenon. Recent studies have suggested that the angiogenic potential of a tumour may be inferred by the vascularity measured in histological sections. The purpose of this study has been to determine whether vascularity is related to age, survival or other clinical parameters in resected non-small-cell lung cancer (NSCLC). A group of 88 consecutive patients with a follow-up period of at least 5 years was selected. The group exhibited a wide age range (37–78 years) and similar survival characteristics to those of the general NSCLC population. Tumour sections were stained with a pan-endothelial antibody (vWF) and vascularity was quantitated, without knowledge of the clinical details, by three methods: highest microvascular density; average microvascular density; and average microvascular volume. The results were analysed by non-parametric statistical tests. A correlation was found between all three methods of quantitation. Vascularity was not associated with age, sex, tumour type, stage, volume, size (TNM-T) nodal status (TNM-N) or survival. However, survival time was generally longer for patients with higher vascularity, reaching borderline significance ($P = 0.06$) for the average microvascular density values. Higher tumour volume ($P = 0.02$) and stage ($P = 0.05$) were associated with lower survival times. Using multivariate survival analysis, tumour volume was the only factor related to survival. We conclude that vascularity is not associated with age and has no significant prognostic value in NSCLC.

**Keywords:** lung cancer; vascularity; microvasculature; age; tumour volume; prognosis

The incidence of squamous cell lung cancer increases with age, accounting for approximately 40% of all lung cancers by the age of 80 years (Bryne and Carney, 1995). Clinical studies have indicated that lung carcinomas in the elderly show reduced growth rate and metastatic potential in comparison with those of younger patients (Ershler et al, 1993; Holmes 1989). This is consistent with the finding of an inverse relationship between stage and age in non-small-cell lung cancer (O'Rouke et al, 1987; Teeter et al, 1987).

The formation of new blood vessels, or angiogenesis, has been shown to be essential for tumour growth and metastasis in experimental animal models (Folkman et al, 1990). In such experimental models both angiogenic potential and tumour growth are reduced with age (Yuhas et al, 1974; Kim et al, 1989; Kreisle et al, 1990; Ershler et al, 1993). It is therefore possible that angiogenic suppression may play a part in the reduced growth and metastatic potential observed in lung tumours of elderly patients (Ershler et al, 1993).

Intratumoral microvessels can be easily visualized in histological sections using immunostaining with endothelium-specific antibodies. The stained vessels can then be counted by a variety of methods, the most popular of which is the measurement of the highest microvascular density or 'hotspot' (Weidner 1993). A large number of studies have found the highest microvascular density value to be associated with metastasis and poor survival in various types of tumours (reviewed by Gasparini, 1994; Craft and Harris, 1994). These results suggest, therefore, that the angiogenic potential of a tumour may be inferred by its vascularity. This hypothesis is widely accepted, so that vascularity measured in histological sections using pan-endothelial antibodies is increasingly referred to as 'angiogenesis'. The validity of this hypothesis, however, is questioned by results of several studies that found either no association between vascularity and prognosis (Hall et al, 1992; van Hoef et al, 1993; Axelsson et al, 1995; Busam et al, 1995; Tahan and Stein, 1995; Morphopoulos et al, 1996) or highest vascularity associated with good prognosis (Kainz et al, 1995; Zatterström et al, 1995).

A small number of reports concerning the association between vascularity, metastasis and survival in non-small-cell lung cancer have been published. The results are also divided along the same lines as described above, i.e. the majority of studies found high vascularity to be associated with poor prognosis (Macchiarini et al, 1992; Yamazaki et al, 1994; Fontanini et al, 1995; Yuan et al, 1995; Giatromanolaki et al, 1996), although one large study (comprising over 200 patients) found that vascularity had no prognostic value (Mattern et al, 1995).

The objectives of our study have been to determine whether vascularity in resected non-small-cell lung cancer is associated with either survival or age. It has previously been suggested that contradictory results in the literature may be explained by variations in vascularity among blocks taken from the same tumour (van Hoef et al, 1993; De Jong et al, 1995) and by the methods used to measure vascularity (Pazouki et al, 1997). As quantitative methods in diagnostic pathology must be reliable and straightforward, we have attempted to clarify this situation by measuring vascularity in a well-defined group of NSCLC patients, using three different methods. The patients exhibited a wide age range and similar survival characteristics to those from a larger series previously reported and from the general NSCLC population (Jefferson et al, 1996).

*Received 20 January 1997*
*Revised 14 April 1997*
*Accepted 11 May 1997*

*Correspondence to:* AM Schor

### MATERIALS AND METHODS

### Patients and specimens

A group of 88 patients was selected from a larger series of surgical resections for NSCLC performed at the Cardiothoracic Centre, Liverpool, UK (Jefferson et al, 1996). The patients were chosen from consecutive operative resections by the following criteria: (a) complete survival data were available for a follow-up period of at least 5 years; (b) histological material was available in the form of at least four formalin-fixed, paraffin-embedded blocks; (c) only the most common NSCLC cell types, squamous cell or adenocarcinomas, would be included; and (d) the number of patients had to be large enough so that the group would show survival characteristics representative of the larger series. Although part of the aims of the study was to examine the relationship between age and vascularity, the group chosen by the above criteria had a sufficiently wide age range (37–78 years), so that no other selection criteria were used. All patients underwent surgery in 1988. They were classified by tumour cell type, UICC tumour stage and TNM staging system (T and N) according to published criteria (WHO, 1982; Hermanek and Sobin, 1987; Mountain, 1993).

From each case, a minimum of four blocks were sectioned and reviewed by a pathologist (DMC) in order to confirm the diagnosis and select one block per tumour for the analysis of vascularity. Blocks were selected on the basis of (i) being representative of the tumour, (ii) containing ample tumour areas and (iii) including tumour free margins.

### Immunocytochemistry

Lung tumour sections were dewaxed in xylene, rehydrated in ethanol and finally washed in distilled water for 5 min followed by phosphate-buffered saline (PBS) for 10 min. Endogenous peroxidase was blocked by incubating the sections in 3% hydrogen peroxide in PBS for 10 min. Sections were then pretreated with protease XXIV (Sigma) 1 mg $ml^{-1}$ in PBS at 37 °C for 30 min. This was found to be the optimum antigen retrieval method for lung tissue. Goat serum (20%) in PBS was used to block non-specific sites for 20 min, the excess serum removed and the primary antibody applied. Blood vessels were localized by immunostaining 5 mm-thick sections with rabbit anti-human von Willebrand factor (vWF) antibody (Dako. cat. no. A0082) diluted 1:4000 in 20% normal goat serum (NGS). Sections were incubated with the primary antibody at 4°C overnight and then with the secondary antibody (biotinylated polyclonal goat anti-rabbit IgG diluted 1:166 in 20% NGS) for 30 min. The slides were then incubated with the Vectastain ABC-peroxidase reagent from the Elite amplification kit (Vector cat. no. 6100) for 30 min. Next, they were washed in PBS for 10 min and then incubated in a freshly prepared diaminobenzidine substrate (0.08 g in 200 ml PBS/ 200 µl 3% hydrogen peroxide) for 4 min. Sections were rinsed in PBS, counterstained with haematoxylin, dehydrated and mounted. In preliminary experiments, the antibody to CD31 (clone JC/70A from Dako) was compared to the anti-vWF.

### Assessment of vascularity

Two non-consecutive sections were immunostained per block. In all cases, both histological features and vascularity were similar in the duplicate sections. Vascularity was assessed by light microscopy using three different methods of quantitation and without prior knowledge of patient clinical parameters. These methods were:

1. Highest microvascular density (h-MVD). Any cell or cell cluster showing antibody staining was considered to be a countable microvessel, irrespective of whether or not a lumen was present. Microvessels in sclerotic or necrotic areas within the tumour or those in adjacent areas of unaffected lung parenchyma were not considered in the vessel count. The area of highest microvascular density was located by scanning the section at 120 × magnification. Microvessels were counted at 200 × magnification using a grid that circumscribed an area of 0.476 $mm^2$. All vessels contained either completely or partly within the grid were included in the count. Three separate fields were counted and the highest of these three counts was taken as the h-MVD and expressed as the number of vessels per $mm^2$ (Weidner, 1993).
2. Average microvascular density (a-MVD). Using the same criteria, grid and magnification as above, the number of microvessels were counted in 18 fields selected randomly throughout the section. a-MVD was determined by calculating the mean of the 18 values and expressed as the number of vessels per $mm^2$ ± standard deviation.
3. Microvascular volume (MVV). The MVV was calculated by the conventional stereological method of point counting using an eyepiece graticule that contained 100 points. Vessels that coincided with the points were counted in 18 fields selected randomly across each section (a total of 1800 points) and the results were expressed as percentage volume, taking the mean ± standard deviation of the 18 values.

All specimens were counted by one observer (LMC) after a period of training and assessment of the reliability of the counting methods. This included counting the same (10–15) specimens on separate occasions (a) in conjunction with two other independent observers, using a two-headed microscope, (b) by the first observer alone and (c) by two observers independently.

### Calculation of tumour volume

Measurements of the maximum tumour diameter in three dimensions were available for 51 patients. These three values were multiplied to give the 'actual tumour volume'. As often only one dimension per tumour is available, an 'estimated tumour volume' was calculated by cubing the largest tumour dimension. Both actual and estimated tumour volumes were used in the analyses.

### Statistical analyses

As vascularity data were not normally distributed, statistical analyses were performed by non-parametric tests. Kaplan–Meier (product estimates) and log-rank tests were used for individual variables and Cox's proportional hazards model was used for multivariate survival analysis. Details of the various tests used are given under the appropriate experimental sections. All analyses were carried out using SPSS software (version 6.0, SPSS. Chicago, IL, USA).

© Cancer Research Campaign 1997

**Figure 1** Sections of non-small-cell lung carcinomas stained with vWF antibody. (**A** and **B**) Adenocarcinomas of low and high vascularity respectively. (**C** and **D**) Squamous cell carcinomas of low and high vascularity respectively. Bar = 100 μm for C and 50 μm for A, B and D

### RESULTS

### Patients

Patient characteristics, shown in Table 1, fell within those of the general population of surgically treated NSCLC (Jefferson et al, 1996). Of the 88 patients, 67% were men, 75% had squamous cell carcinoma, 57% presented with stage I and 63% without bronchial or mediastinal nodal involvement. At the time of the analyses, 53 patients were dead and 35 were alive.

### Histological features: microvessel staining and distribution

The protocol adopted to immunostain blood vessels was optimized using nine specimens of NSCLC and two pan-endothelial antibodies: vWF and CD31. Using the optimal antibody concentration and incubation times, we found significant differences in the number of vessels stained depending on the pretreatment of the sections. Under all conditions examined we found vWF preferable to CD31 regarding the intensity of staining and number of vessels detected (results not shown). All subsequent staining was performed with vWF antibody.

Using the optimal protocol (as described in Materials and methods), immunostaining with vWF was satisfactory for all the sections examined and variations in the intensity of the staining were not evident. The distribution and density of the vessels were consistent for the duplicate sections of a given block, but great variation was observed among different tumours. For example, vessels could be either homogeneously or heterogeneously distributed throughout the section, the highest vessel density could be at the periphery of the tumour or towards the centre. These variations were found for both adenocarcinomas and squamous cell carcinomas. Examples of high and low vascularity for both tumour types are shown in Figure 1.

It has been reported previously that the clinical outcome of oral squamous cell carcinoma patients appears to be correlated with different patterns of tumour architecture and blood supply (Lauk et al, 1989). Therefore, for each tumour we assessed the position of the highest vascularity area (hotspot). In addition, we graded the following features: (i) vascularity throughout the tumour; (ii) vascularity at the edge of the tumour; (iii) lymphocyte infiltration; and (iv) tumour architecture. None of these parameters was related to patient outcome, as determined by the chi-square test (results not shown).

### Correlation between three methods of quantitating vascularity

Using the Spearman or Kendall correlations, all three measurements of vascularity were found to be significantly associated ($P$<0.0001) (Figure 2). The highest correlation occurred between the two methods that measure density (h-MVD and a-MVD, $r$=0.78) and the lowest correlation was between the highest density and the volume (h-MVD and MVV, $r$ = 0.52).

### Relationship between vascularity, age and other clinical parameters

Clinicopathological characteristics of the patients and the vascularity of the tumours are presented in Table 1. Applying the Wilcoxon rank-sum test, tumour vascularity, assessed by any of the three methods used, was not associated with age, sex, tumour type, volume, stage, size (TNM-T), nodal status (TNM-N) or patient outcome. Similarly, age was not associated with any of the parameters examined (Table 1).

### Analysis of survival

Assessment of the association between vascularity, age and other clinical parameters with overall survival was investigated by the Kaplan–Meier estimates of survival function, with comparisons made using the log-rank tests (Table 2). The median values were calculated for all three measures of vascularity (4.4 for MVV, 140.8 for h-MVD, 67.2 for a-MVD), as well as for age (65 years),

© Cancer Research Campaign 1997

**Figure 2** Correlation between different methods of quantitating vascularity. The methods compared were: microvascular volume (MVV), highest microvascular density (h-MVD) and average microvascular density (a-MVD). Correlation coefficients ($r$) were as follows: (**A**) a-MVD v h-MVD $r = 0.78$; (**B**) MVV v h-MVD $r = 0.52$; (**C**) MVV v a-MVD $r = 0.58$. $P<0.001$ for all groups

'actual' tumour volume (36 $cm^3$) and 'estimated' tumour volume (64 $cm^3$). These values were used as cut-off points for the analyses. Survival time was significantly associated with tumour stage ($P$ = 0.05) and tumour volume (both actual, $P = 0.02$ and estimated, $P$ = 0.003). Two other parameters were associated with survival at a 93% or 94% level of confidence; these were nodal status ($P$ = 0.07) and vascularity measured by the a-MVD method ($P = 0.06$). Survival was not associated with any of the other parameters examined, including age and vascularity measured by either h-MVD or MVV (Table 2). Survival curves according to median values of the three vascularity measurements are shown in Figure 3. It should be noted that the association between a-MVD and survival indicated that high vascularity was associated with good prognosis (Table 2 and Figure 4C).

A multivariate survival analysis was performed with the two variables found to be individually related to survival time (tumour volume and stage). In this model, tumour stage ceased to be predictive of survival; tumour volume was the only measured factor related to survival, with a 58% (95% CI 52–65%) relative increase in hazard for tumours larger than 64 $cm^3$ ($P$=0.005). Applying the Kruskal–Wallis test, higher tumour volumes were significantly associated with greater levels of nodal status (TNM-N) ($P$<0.05) Survival curves according to tumour volume defined by median groupings are shown in Figure 4.

### DISCUSSION

This study was designed to determine whether vascularity in resected NSCLC was associated with patient survival or age. Accordingly, the patients were selected from a larger series previously examined (Jefferson et al, 1996) so that their outcome was representative of that of the larger series, a prolonged follow-up period was available and a wide range of ages was included. As with the general population of surgically treated NSCLC, the 88 patients that we examined fell into two distinct groups with either high or low median survival. We found that vascularity was not significantly associated with age, survival or any other clinical parameter, including tumour type, volume, stage, size (TNM-T) or nodal status (TNM-N).

It is interesting that survival time was generally longer for patients with higher vascularity values, but this association only reached borderline significance for a-MVD, one of the three vascularity measurements used (Table 2 and Figure 3). Several studies have reported that high microvascular density is a good prognostic indicator in carcinoma of the cervix (Siracka et al, 1988; Revesz et al, 1989; Kainz et al, 1995), and head and neck tumours (Zatterström et al, 1995). However, the majority of studies have found the opposite, i.e. that high vascularity is an indicator of poor prognosis (see Introduction).

To date, all studies measuring vascularity in NSCLC have counted the vessels in the most vascularized area or 'hotspot' either as density (h-MVD) (Macchiarini et al, 1992; Fontanini et al, 1995; Yuan et al, 1995) or as point counting a total of 75 points (Giatromanolaki et al, 1996). A good correlation has been found between different methods to measure vascularity, including subjective eye appraisal (Giatromanolaki et al, 1996). However, we have previously reported that different results can be obtained when vascularity is estimated by the microvascular volume, rather than density (Pazouki et al, 1997). Therefore, in this study we have assessed vascularity by three different methods: two that measure density, either randomly (a-MVD) or in the 'hotspot' (h-MVD), and one that estimates volume by stereological point counting throughout the section (MVV). Apart from the near-significant association with survival discussed above, the three measurements of vascularity showed the same lack of association with age, survival and clinicopathological parameters (Tables 1 and 2 and Figure 3).

© Cancer Research Campaign 1997

**Table 1** Vascularity, age and other patient characteristics

| Variable | Number of cases | MVV median (IQR) | h-MVD median (IQR) | a-MVD median (IQR) | Age median (IQR) |
|---|---|---|---|---|---|
| All | 88 | 4.4 (3.3–5.5) | 140.8 (103–192) | 67.2 (47.3–98.7) | 65 (61–68) |
| Age | | | | | |
| <65 years | 46 | 4.1 (3.3–5.8) | 143 (103–183) | 67.2 (56.7–84) | – |
| >65 years | 42 | 4.3 (3.5–5.4) | 139 (103–212) | 65.1 (44.1–105) | – |
| Sex | | | | | |
| Male | 59 | 4.2 (3.5–5.4) | 135 (99–181) | 65.1 (44.1–98.8) | 66 (61–69) |
| Female | 29 | 4.3 (3.4–5.8) | 145 (116–202) | 67.2 (54.6–88.2) | 63 (60–68) |
| Tumour type | | | | | |
| Squamous cell | 66 | 4.3 (3.4–6.3) | 144 (103–202) | 67.2 (50.4–105) | 65 (61–68) |
| Adenocarcinoma | 22 | 3.7 (2.8–4.7) | 125 (105–160) | 67.2 (44.1–84) | 64 (63–69) |
| Actual tumour volume | | | | | |
| <36 cm³ | 27 | 4.3 (3.7–5.5) | 147 (116–190) | 75.6 (58.8–96.6) | 63 (57–67) |
| >36 cm³ | 24 | 4.6 (3.2–5.9) | 149 (88–234) | 67.2 (44.1–120.8) | 66 (62–70) |
| Estimated tumour volume | | | | | |
| <64 cm³ | 30 | 4.3 (3.8–5.8) | 152 (116–200) | 77.7 (60.9–111.3) | 63 (58–70) |
| >64 cm³ | 21 | 4.6 (2.7–5.5) | 135 (86–216) | 67.2 (37.8–107.1) | 67 (62–69) |
| Tumour stage (UICC) | | | | | |
| I | 50 | 4.3 (3.2–5.3) | 139 (103–185) | 67.2 (44.1–90.3) | 64 (61–70) |
| II | 14 | 3.5 (2.4–4.3) | 126 (111–183) | 60.9 (37.8–111.3) | 66 (59–68) |
| IIIa | 24 | 4.6 (3.6–6.5) | 145 (105–206) | 66.2 (53.6–98.7) | 64 (63–68) |
| TNM-T | | | | | |
| T1 | 32 | 4.2 (2.8–5) | 145 (103–160) | 68.2 (44.1–100.8) | 63 (57–68) |
| T2 | 46 | 4.4 (3.2–5.5) | 135 (101–193) | 67.2 (50.4–84) | 65 (62–68) |
| T3 | 10 | 4.3 (3.7–6.5) | 191 (110–215) | 67.2 (50.4–107.1) | 66 (61–71) |
| TNM-N | | | | | |
| N0 | 55 | 4.3 (3.5–5.3) | 143 (103–193) | 67.2 (47.3–99.8) | 64 (61–71) |
| N1 | 17 | 3.8 (2.5–5.9) | 117 (108–181) | 63 (37.8–94.5) | 66 (59–68) |
| N2 | 16 | 4.6 (3.7–5.5) | 147 (105–204) | 65.1 (56.7–92.4) | 64 (63–67) |
| Outcome | | | | | |
| Dead | 53 | 3.9 (3.3–5.8) | 135 (99–191) | 60.9 (44.1–84) | 64 (61–69) |
| Alive | 35 | 4.3 (3.6–5.2) | 147 (112–197) | 77.7 (55.7–112.4) | 65 (60–68) |

Vascularity measurements were: microvascular volume (MVV, as %); highest microvascular density (h-MVD, as vessels $mm^{-2}$); and average microvascular density (a-MVD, as vessels $mm^{-2}$). Age is given in years. IQR = inter-quartile range.

Our results disagree with other studies reporting that h-MVD in NSCLC was significantly associated with histological type, stage of disease (Yuan et al, 1995), nodal status (Fontanini et al, 1995; Yuan et al, 1995; Giatromanolaki et al, 1996) distant metastasis (Macchiarini et al, 1992; Fontanini et al, 1995; Yuan et al, 1995; Giatromanolaki et al, 1996) relapse free and overall survival (Yamazaki et al, 1994; Fontanini et al, 1995; Giatromanolaki et al, 1996). In contrast, our results agree with those of Mattern et al (1995), who found that h-MVD was not a prognostic indicator for metastasis or survival in NSCLC; similarly, Yamazaki et al (1994) found that vascularity was not associated with lymph node metastasis in adenocarcinomas.

Established prognostic indicators in NSCLC include nodal involvement, disease stage and tumour grade (Shimosato, 1995). Recently, a clinical study reviewing over 600 patients found that tumour volume was associated with survival (Jefferson et al, 1996). In spite of the small number of tumours examined in our study, we found that larger tumour volumes were associated with shorter survival times ($P<0.02$), as well as with nodal status (TNM-N) ($P<0.05$). It is interesting to note that both 'actual' and 'estimated' tumour volumes correlated with survival, allowing the possibility of using estimated tumour volumes in calculations of survival when only the largest tumour dimension is available. Our study also confirms that tumour stage is significantly associated with survival, whereas the association between nodal involvement and survival only reached borderline significance.

In our study, age was not associated with vascularity or with any other of the parameters tested, including survival. In contrast, an association between age and vascularity has been found in NSCLC (Giatromanolaki et al, 1996) and other types of tumours (Davidson et al, 1994; Miliaras et al, 1995; Leon et al, 1996). When reported, tumours from younger patients had higher vascularity values than those from older patients. However, several studies have been unable to confirm the statistical significance of this association (van Hoef et al, 1993; Axelsson et al, 1995; Bossi et al, 1995; Tahan and Stein, 1995). A lack of association between age and other prognostic indicators has previously been reported for NSCLC (Pendleton et al, 1996), oral carcinoma (Williams et al, 1994) and cutaneous melanoma (Busam et al, 1995).

The estimated vascularity in tissue sections can be significantly affected by variations in the methodology, including antibody used and pretreatment of the sections (Busam et al, 1995; our

© Cancer Research Campaign 1997

**Table 2** The association between vascularity, age and other clinical parameters with survival

| Variable group (number of cases) | Median survival in days (95% CI) | Probability of survival (%) between 2 and 5 years | | Significance of relationship to survival time |
|---|---|---|---|---|
| All patients (88) | 994 (142–1845) | 48.9 | 40.9 | – |
| Age | | | | |
| <65 years (46) | 535 (0–1967) | 50 | 40 | $P = 0.79$ |
| >65 years (42) | 994 (0–2756) | 47.6 | 45.2 | |
| Sex | | | | |
| Male (59) | 751 (252–1250) | 50.9 | 37.3 | $P = 0.47$ |
| Female (29) | 2145 (597–3692) | 58.6 | 48.3 | |
| Tumour type | | | | |
| Squamous cell (66) | 1005 (0–2334) | 50 | 42.4 | $P = 0.88$ |
| Adenocarcinoma (22) | 592 (0–1522) | 45.5 | 36.4 | |
| MVV | | | | |
| <4.4 (46) | 810 (0–2344) | 52.1 | 41.3 | $P = 0.74$ |
| >4.4 (42) | 1224 (274–2173) | 52.4 | 40.5 | |
| h-MVD | | | | |
| <140.8 (45) | 751 (324–1178) | 51.1 | 35.6 | $P = 0.35$ |
| >140.8 (43) | 1498 (*,*) | 58.1 | 46.5 | |
| a-MVD | | | | |
| <67.2 (48) | 592 (163–1021) | 45.8 | 33.3 | $P = 0.06$ |
| >67.2 (40) | 2145 (*,*) | 65 | 50 | |
| Actual tumour volume | | | | |
| <36 cm³ (27) | 2613 (*,*) | 66.7 | 55.6 | $P = 0.02$ |
| >36 cm³ (24) | 355 (104–606) | 25 | 25 | |
| Estimated tumour volume | | | | |
| <64 cm³ (30) | 2651 (*,*) | 66.7 | 56.7 | $P = 0.003$ |
| >64 cm³ (21) | 348 (298–412) | 19.1 | 19.1 | |
| Tumour stage (UICC) | | | | |
| I (50) | 2230 (*,*) | 60 | 52 | $P = 0.05$ |
| II (14) | 810 (0–1861) | 57.1 | 35.7 | |
| IIIa (24) | 565 (227–904) | 33.3 | 20.8 | |
| TNM-T | | | | |
| T1 (32) | 2230 (*,*) | 65.6 | 53.1 | $P = 0.3$ |
| T2 (46) | 565 (22–1108) | 40 | 35.6 | |
| T3 (10) | 643 (12–1274) | 36.4 | 27.3 | |
| TNM-N | | | | |
| N0 (55) | 2145 (*,*) | 58.2 | 49 | $P = 0.07$ |
| N1 (17) | 614 (0–1377) | 31.3 | 31.3 | |
| N2 (16) | 205 (163–967) | 35.3 | 23.5 | |

Medians were used as cut-off values for vascularity measurements (MVV, h-MVD, a-MVD), age and tumour volumes. 95% CI represents the 95% confidence intervals; when they are not calculable *,* is entered. The significance of association with survival is from the log-rank test.

unpublished data). Even after careful optimization of the methodology, heterogeneity among blocks taken from the same tumour can significantly affect the microvascular density attributed to a given tumour (van Hoef et al, 1993; De Jong et al, 1995; Schor et al, manuscript in preparation). We suggest, therefore, that variations in methodology and tumour heterogeneity could account for the contradictory reports regarding the value of vascularity as a tumour prognostic indicator. In a large tumour, such as most resected NSCLC, it may be necessary to examine multiple blocks in order to determine the overall vascularity of the tumour.

Vascularity, estimated using pan-endothelial antibodies such as vWF or CD31, is often referred to as angiogenesis; however, these antibodies do not distinguish between newly formed (angiogenic) and pre-existing vessels. Furthermore, as microvascular density in normal bronchial tissue is similar (h-MVD) or higher (a-MVD) than that of lung tumours (Schor et al, manuscript in preparation), there is no evidence that vascularity measured in these tumours by staining the vessels with a pan-endothelial marker represents angiogenesis. Specific anti-angiogenic vessel antibodies for paraffin-embedded tissues are not yet available.

Taken together, our results indicate that vascularity, measured by either density or volume of vessels stained with pan-endothelial markers, is (a) not a prognostic indicator in NSCLC and (b) not related to the age of the patients. Our results do not exclude the possibility that angiogenesis may be important in lung tumours, but suggest that, if this is the case, vascularity measured by current methods is not an accurate index of angiogenesis in these tumours.

© Cancer Research Campaign 1997

**Figure 3** Survival curves according to median vascularity values. Vascularity measurements were: (**A**) microvascular volume (MVV, as %) <4.4, ——; >4.4, ------; (**B**) highest microvascular density (h-MVD, as vessels mm$^{-2}$) <140.8, ——; >140.8, ------; (**C**) average microvascular density (a-MVD, as vessels mm$^{-2}$) <67.2, ——; >67.2, ------.

**Figure 4** Survival curves according to median tumour volumes. Tumour volume measurements were: (**A**) actual tumour volume divided into those <36 $cm^3$ (——), >36 $cm^3$ (-------); (**B**) estimated tumour volume divided into <64 $cm^3$ (——), >64 $cm^3$ -------

### ACKNOWLEDGEMENTS

We thank the Roy Castle Lung Cancer Foundation for financial support; Dr MW Myskow (Department of Histopathology, Cardiothoracic Centre and Broadgreen Hospital, Liverpool) for providing the tissue specimens; Mr G Carmichael and Mrs M Thompson (Department of Dental Surgery and Periodontology, Dundee University) for excellent technical assistance; and Dr C Hou (Ninewells Hospital, Dundee) for statistical analyses.

### REFERENCES

Axelsson K, Ljung BME, Moore DH, Thor AD, Chew KL, Edgerton SM, Smith HS and Mayall BH (1995) Tumour angiogenesis as a prognostic assay for invasive ductal breast carcinoma. *J Natl Cancer Inst* **87**: 999–1008

Bossi P, Giuseppe V, Lee A, Alfano R, Coggi G and Bosari S (1995) Angiogenesis in colorectal tumours: Microvessels quantitation in adenomas and carcinomas with clinicopathological correlations. *Cancer Res* **55**: 5049–5053

Busam KJ, Berwick M, Blessing K, Fandrey K, Kang S, Karaoli T, Fine J, Cochran A, White W, Rivers J, Elder D, Wen DR, Heyman B and Barnhill

*© Cancer Research Campaign 1997*

RL (1995) Tumour vascularity is not a prognostic factor for malignant melanoma of the skin. *Am J Pathol* **147**: 1047–1056

Byrne A and Carney DN (1995) Lung cancer in the elderly. In *Lung Cancer*, Carney DN (ed), pp. 267–273. Arnold: London

Craft PS and Harris AL (1994) Clinical prognostic significance of tumour angiogenesis. *Ann Oncol* **5**: 305–311

Davidson S, Ngan R, Wilks D, Moore J and West C (1994) A comparison of four methods for assessing tumour vascularity in carcinoma of the cervix. *Int J Oncol* **5**: 639–645

De Jong JS, van Diest PJ and Baak JPA (1995) Heterogeneity and reproducibility of microvessel counts in breast cancer. *Lab Invest* **73**: 922–926

Ershler WB (1993) The influence of an aging immune system on cancer incidence and progression. *J Am Geriatr Soc* **48**: B3–B7

Folkman J (1990) What is the evidence that tumours are angiogenesis dependent? *J Natl Cancer Inst* **82**: 4–6

Fontanini G, Bigini S, Vignati S (1995) Microvessel count predicts metastatic disease and survival in non small cell lung cancer. *J Pathol* **177**: 57–63

Gasparini G (1994) Quantification of intratumoural vascularisation predicts metastasis in human solid tumours (review). *Oncol Reports* **1**: 7–12

Giatromanolaki A, Koukourakis M, O'Byrne K, Fox S, Whitehouse R, Talbot DC, Harris AL and Gatter KC (1996) Prognostic value of angiogenesis in operable non small cell lung cancer. *J Pathol* **179**: 80–88

Hall NR, Fish DE, Hunt N, Goldin RD and Guillou PJ (1992) Is the relationship between angiogenesis and metastasis in breast cancer real? *Surg Oncol* **1**: 223–229

Hermanek P and Sobin LH (1987) (eds) *TNM Classification of Malignant Tumours*, 4th edn. Springer: Berlin

Holmes FF (1989) Clinical evidence for a change in tumour aggressiveness with age. *Semin Oncol* **16**: 28–33

Jefferson MF, Pendleton N, Faragher EB, Dixon GR, Myskow MW and Horan MA (1996) Tumour volume as a predictor of survival after resection of non-small cell lung cancer (NSCLC). *Br J Cancer* **74**: 456–459

Kainz C, Speiser P, Wanner C, Obermair A, Tempfer C, Sliutz G, Reinthaller A and Breitenecker G (1995) Prognostic value of tumour microvessel density in cancer of the uterine cervix stage 1B to IIB. *Anticancer Res* **15**: 1549–1552

Kim YT, Schwab R, Siskind GW and Weskler ME (1989) Cellular basis for the slower growth of the B16 melanoma in old mice. *Aging: Immunol Infect Dis* **1**: 237–244

Kreisle R, Stebler BA and Ershler WB (1990) Effect of host age on tumour associated angiogenesis in mice. *J Natl Cancer Inst* **82**: 44–47

Lauk S, Skates S, Goodman M and Suit HD (1989) A morphometric study of the vascularity of oral squamous cell carcinomas and its relation to outcome of radiation therapy. *Eur J Cancer Clin Oncol* **25**: 1431–1440

Leon SP, Folkerth RD and Black PM (1996) Microvessel density is a prognostic indicator for patients with astroglial brain tumors. *Cancer* **77**: 362–372

Macchiarini P, Fontanini G, Hardin MJ, Squartini F and Angeletti CA (1992) Relation of neovascularisation to metastasis of non-small-cell lung cancer. *Lancet* **340**: 145–146

Mattern J, Koomägi R and Volm M (1995) Vascular endothelial growth factor and angiogenesis in non small cell lung cancer. *Int J Oncol* **6**: 1059–1062

Miliaras D, Kamas A and Kalekou H (1995) Angiogenesis in invasive breast carcinoma: is it associated with parameters of prognostic significance? *Histopathology* **26**: 165–169

Morphopoulos G, Pearson M, Ryder WDJ, Howell A and Harris M (1996) Tumour angiogenesis as a prognostic marker in infiltrating lobular carcinoma of the breast. *J Pathol* **180**: 44–49

Mountain CF (1993) Lung cancer staging classification. *Clin Chest Med* **14**: 43–53

O'Rourke MA, Feussner JR, Feigl P and Laszio J (1987) Age trends of lung cancer stage at diagnosis. Implications for lung cancer screening in the elderly. *JAMA* **258**: 921–926

Pazouki S, Chisholm DM, Adi MM, Carmichael G, Farquharson M, Ogden GR, Schor SL and Schor AM (1997) The association between tumour progression and vascularity in the oral mucosa. *J Pathol* **183**: (in press)

Pendleton N, Jefferson MF, Dixon GR, Myskow MW and Horan MA (1996) Correlates of tumour size, gender, cell type, and metastasis of resected non-small cell lung cancer and age. *J Gerontol Biol Sci* **51A**: B50–B53

Revesz L, Siracka E, Siracky J, Delides G and Pavlaki K (1989) Variation of vascular density within and between tumours of the uterine cervix and its predictive value for radiotherapy. *Int J Rad Oncol Biol Phys* **16**: 1161–1163

Shimosato Y (1995) Pathology of lung cancer. Development, progression and function. In *Lung Cancer*, Carney DN (ed), pp. 28–43. Arnold: London

Siracka E, Revesz L, Kovac R and Siracky J (1988) Vascular density in carcinoma of the uterine cervix and its predictive value for radiotherapy. *Cancer* **41**: 819–822

Tahan SR and Stein AL (1995) Angiogenesis in invasive squamous cell carcinoma of the lip: tumour vascularity is not an indicator of metastatic risk. *J Cut Pathol* **22**: 236–240

Teeter SM, Holmes FF and McFarlane MJ (1987) Lung carcinoma in the elderly population. Influence of histology on the inverse relationship of stage to age. *Cancer* **60**: 1331–1336

van Hoef MEHM, Knox WF, Dhesi SS, Howell A and Schor AM (1993) Assessment of tumour vascularity as a prognostic factor in lymph node negative invasive breast cancer. *Eur J Cancer* **29A**: 1141–1145

Weidner N (1993) Tumour angiogenesis: Review of current applications in tumour prognostication. *Sem Diag Pathol* **10**: 302–313

World Health Organization (1982) The World Health Organization Histological typing of lung tumours. *Am J Clin Pathol* **77**: 123–136

Williams J, Carlson G, Cohen C, Derose P, Hunter S and Jurkiewicz MJ (1994) Tumour angiogenesis as a prognostic factor in oral cavity tumours. *Am J Surgery* **168**: 373–380

Yamazaki K, Abe S, Takekawa H, Sukoh N, Wanatabe N, Ogura S, Nakajima I, Isobe H, Inoue K and Kawakami Y (1994) Tumor angiogenesis in human lung adenocarcinoma. *Cancer* **74**: 2245–2250

Yuan Y, Yang PC, Yu CJ, Lee YC, Yao YT, Chen CL, Lee LN, Kuo SH and Luh KT (1995) Tumour angiogenesis correlates with histological type and metastasis in non small cell lung cancer. *Am J Respir Crit Care Med* **152**: 2157–2162

Yuhas JM, Pazimo NH, Procter JO and Toya RE (1974) A direct relationship between immune competence and the subcutaneous growth rate of a malignant murine lung tumour. *Cancer Res* **34**: 722–728

Zatterström UK, Brun E, Willen R, Kjellen E and Wennerberg J (1995) Tumour angiogenesis and prognosis in squamous cell carcinoma of the head and neck. *Head Neck* **17**: 312–318

© Cancer Research Campaign 1997


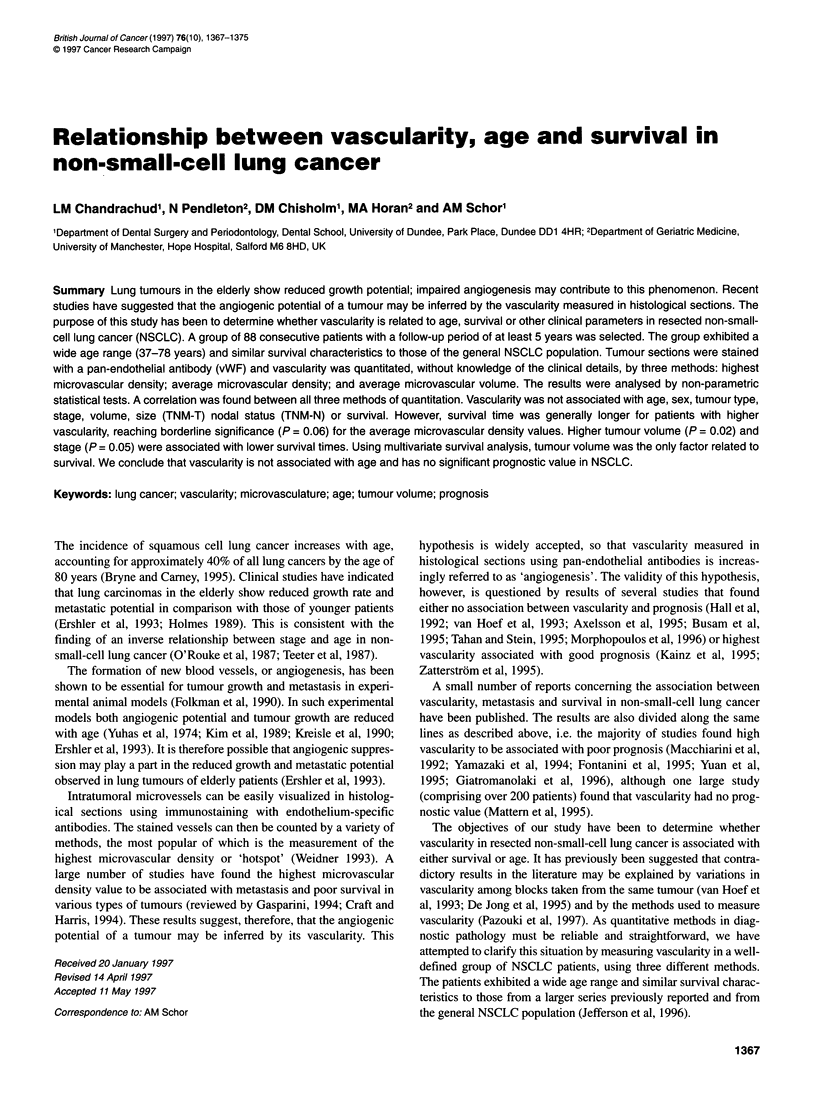

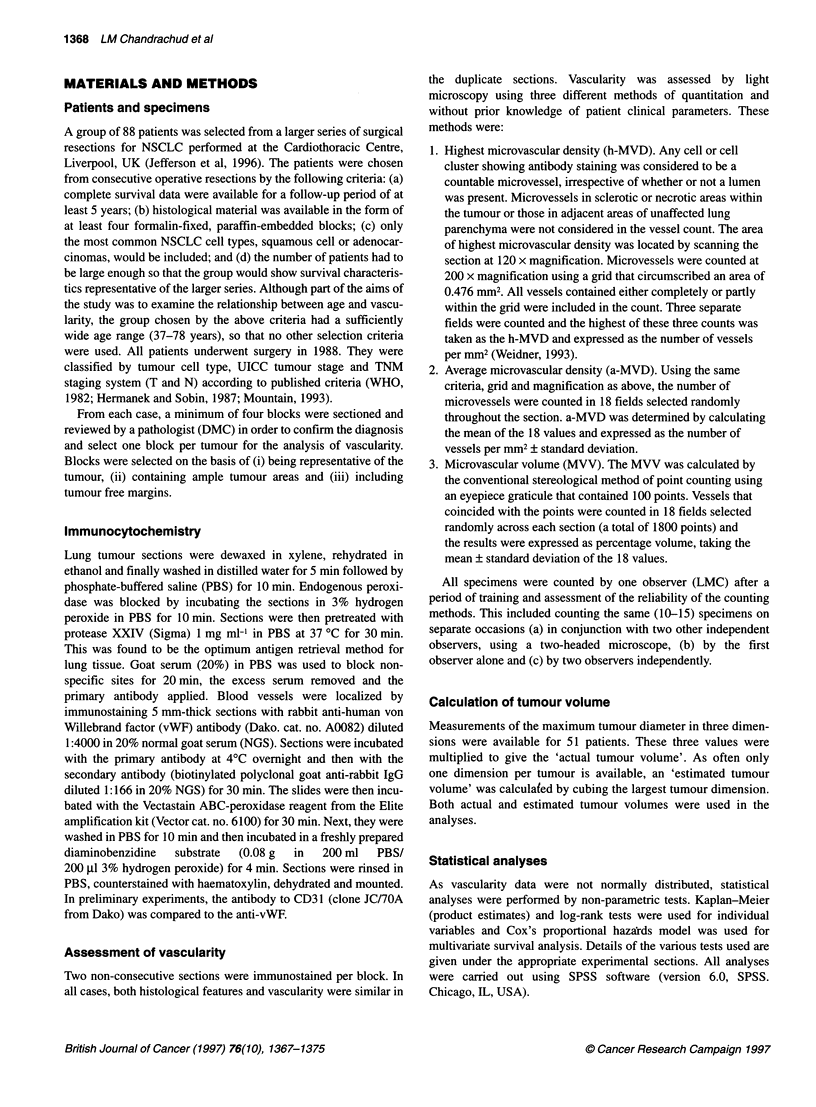

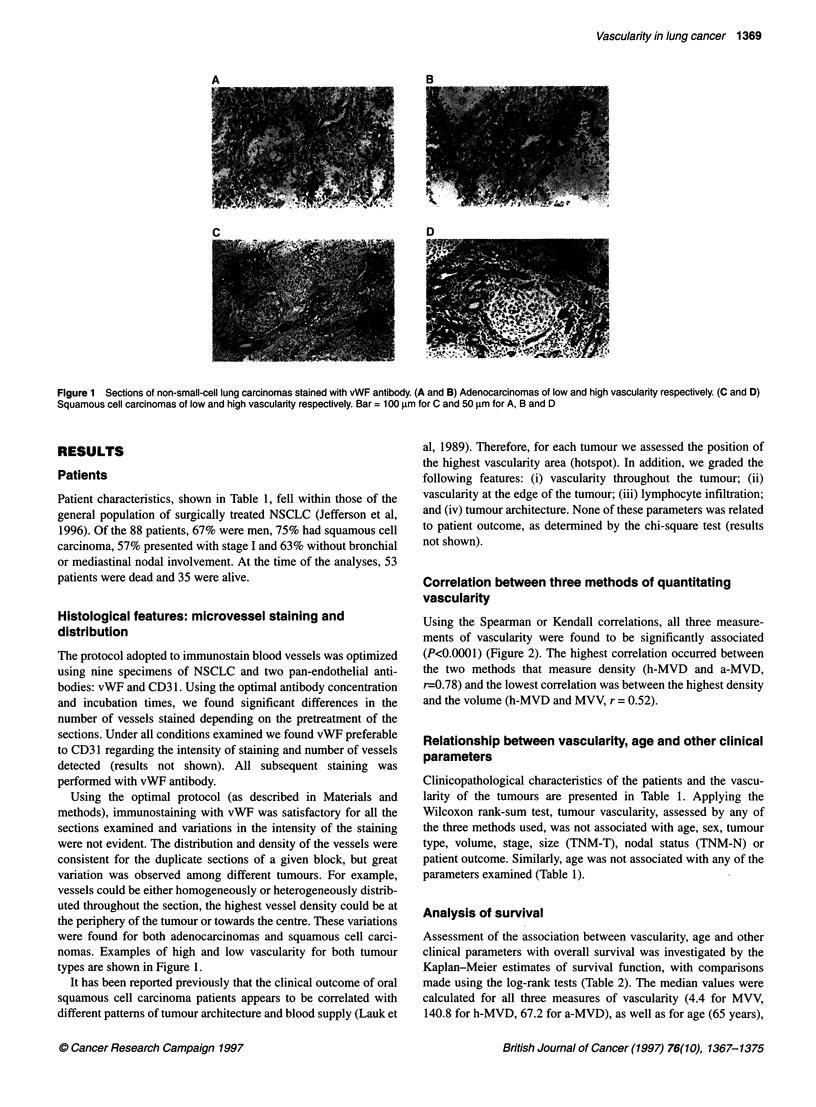

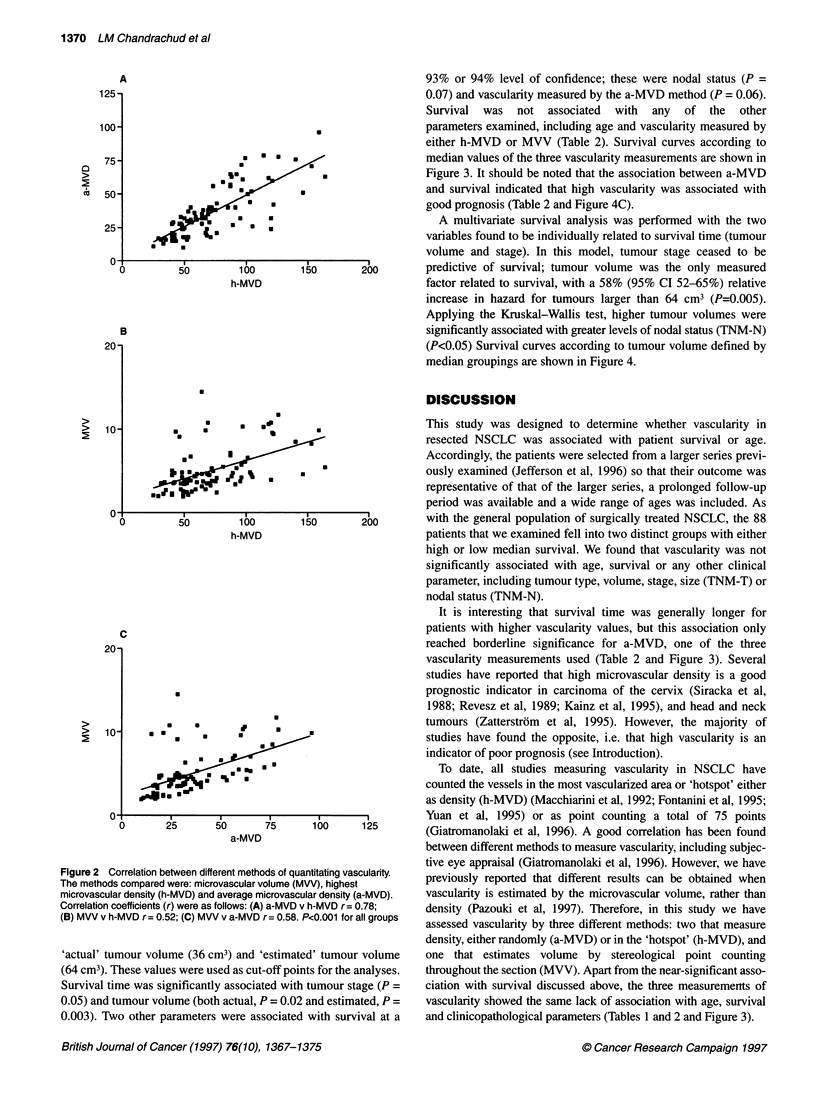

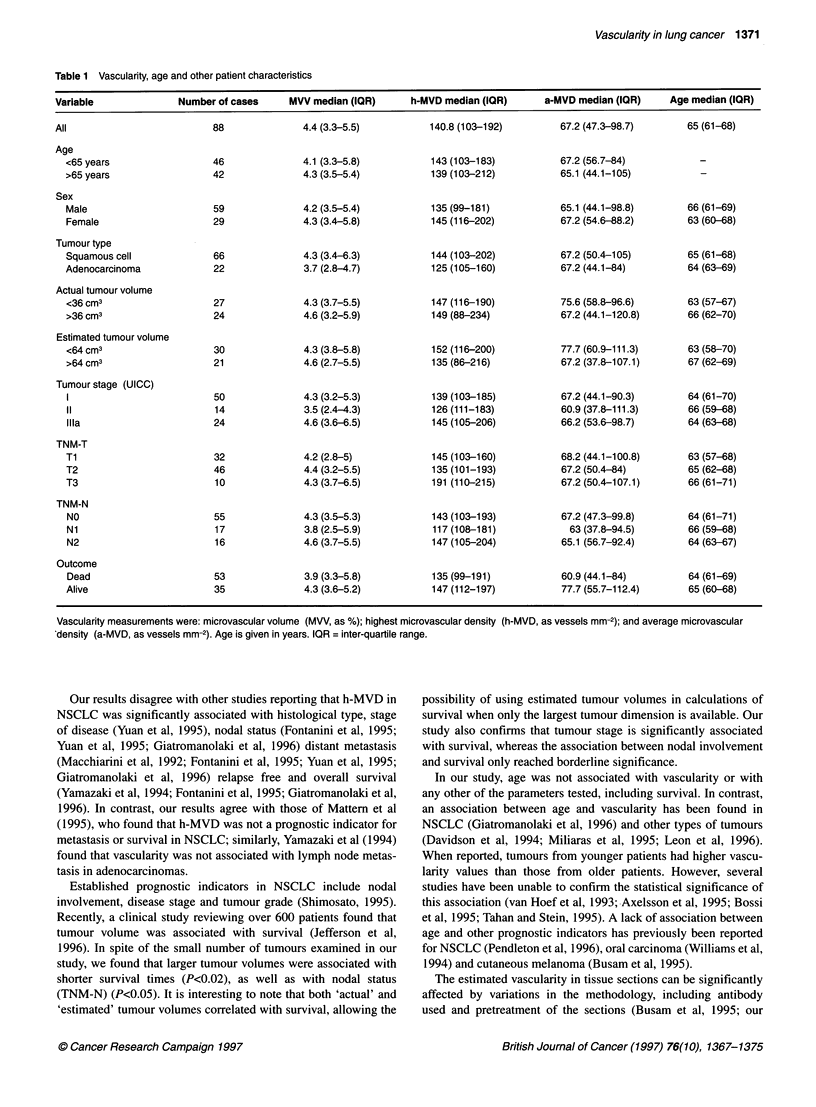

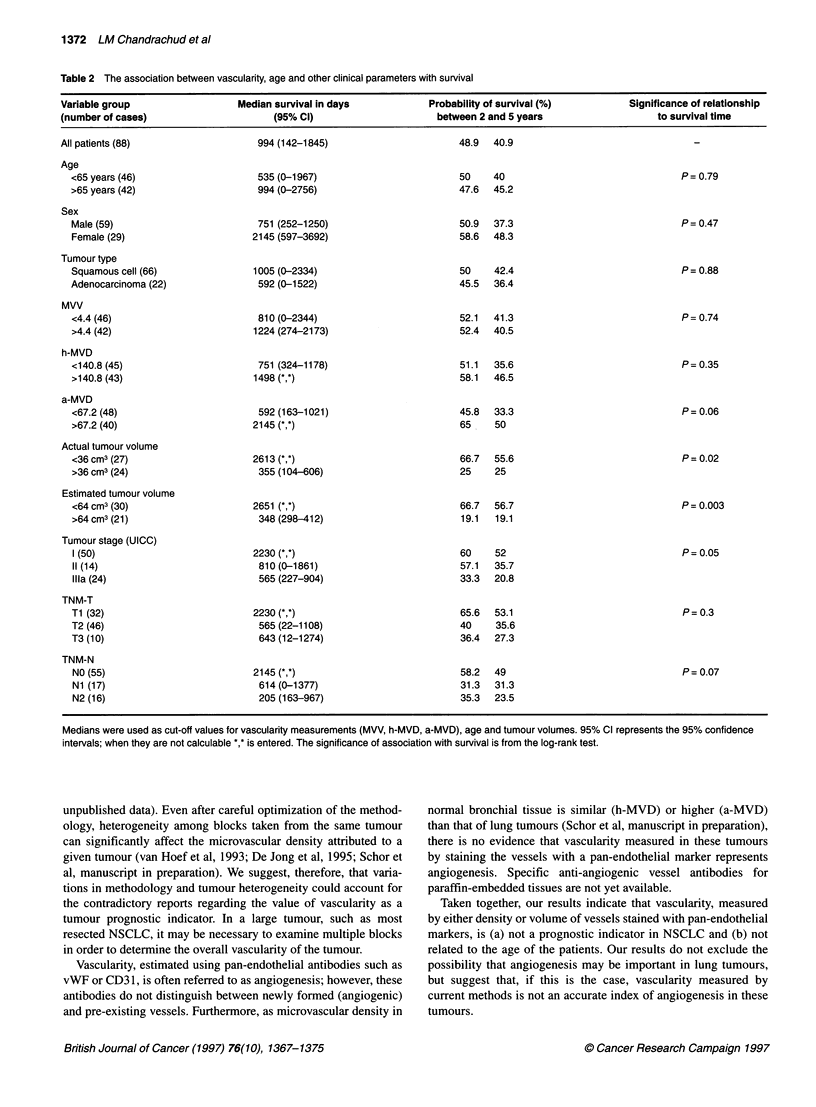

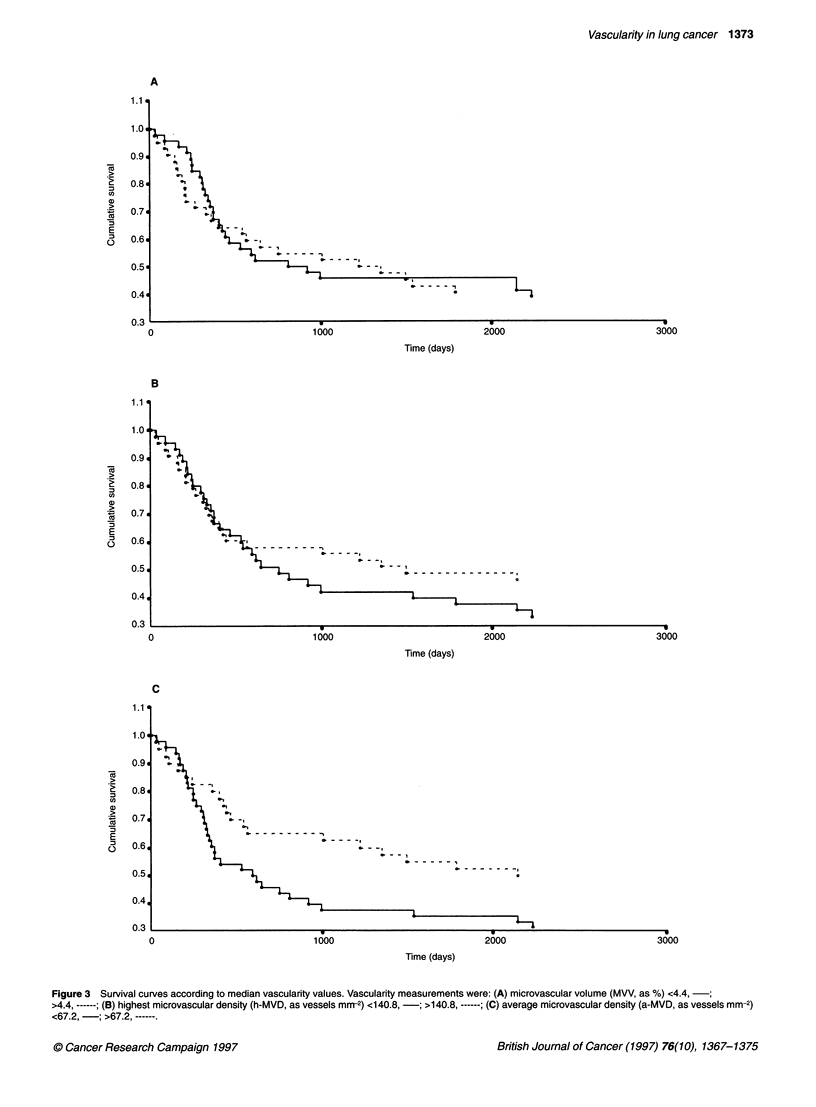

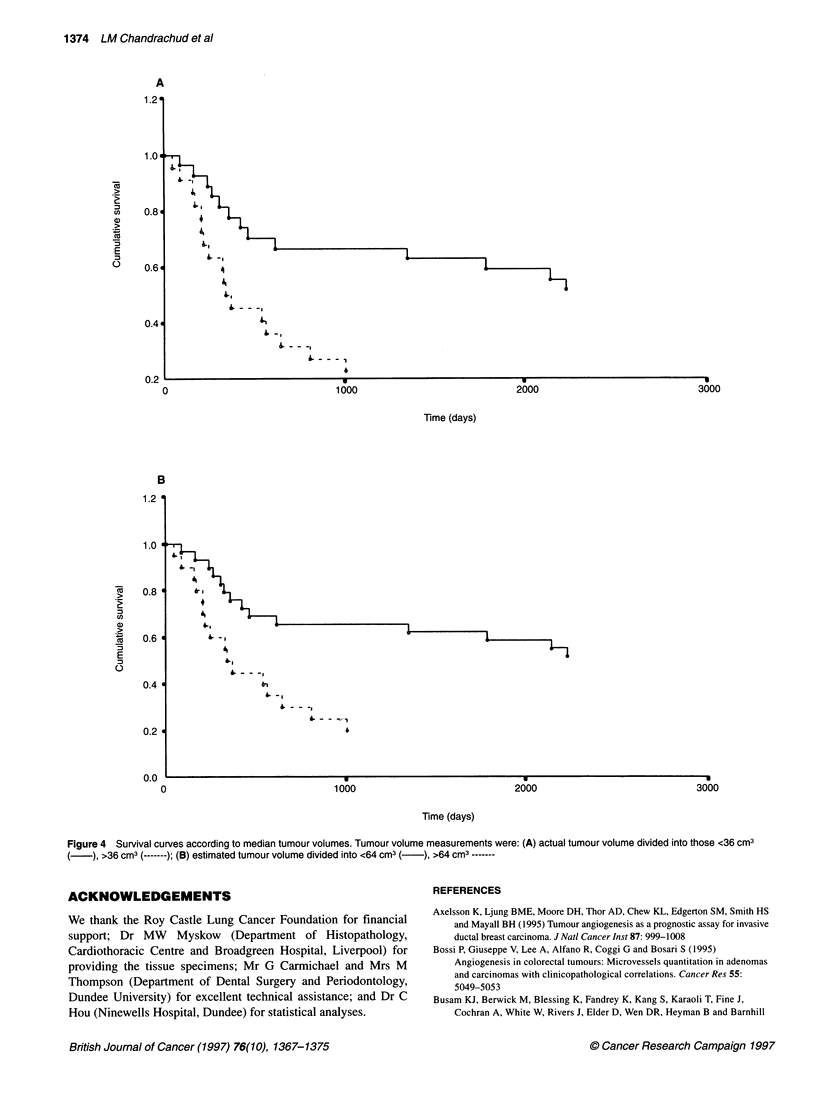

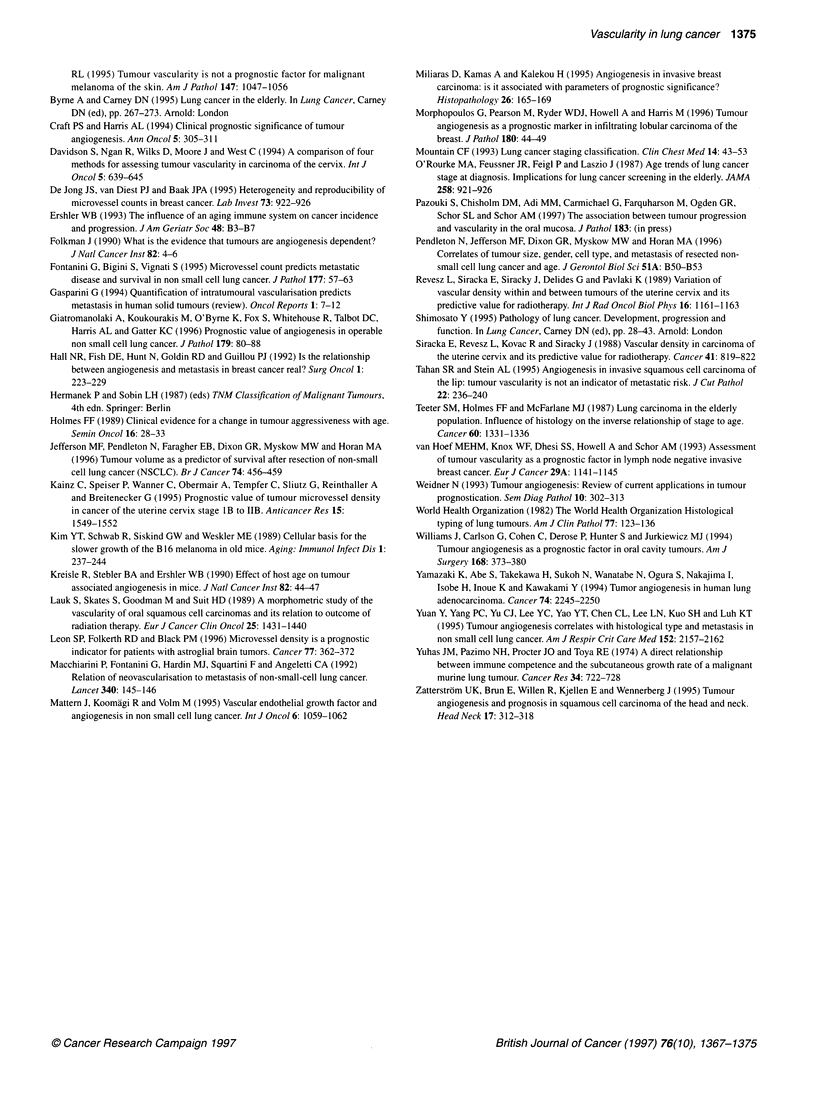


## References

[OCR_00977] Axelsson K., Ljung B. M., Moore D. H., Thor A. D., Chew K. L., Edgerton S. M., Smith H. S., Mayall B. H. (1995). Tumor angiogenesis as a prognostic assay for invasive ductal breast carcinoma.. J Natl Cancer Inst.

[OCR_00982] Bossi P., Viale G., Lee A. K., Alfano R., Coggi G., Bosari S. (1995). Angiogenesis in colorectal tumors: microvessel quantitation in adenomas and carcinomas with clinicopathological correlations.. Cancer Res.

[OCR_01003] Craft P. S., Harris A. L. (1994). Clinical prognostic significance of tumour angiogenesis.. Ann Oncol.

[OCR_01024] Fontanini G., Bigini D., Vignati S., Basolo F., Mussi A., Lucchi M., Chine S., Angeletti C. A., Harris A. L., Bevilacqua G. (1995). Microvessel count predicts metastatic disease and survival in non-small cell lung cancer.. J Pathol.

[OCR_01031] Giatromanolaki A., Koukourakis M., O'Byrne K., Fox S., Whitehouse R., Talbot D. C., Harris A. L., Gatter K. C. (1996). Prognostic value of angiogenesis in operable non-small cell lung cancer.. J Pathol.

[OCR_01036] Hall N. R., Fish D. E., Hunt N., Goldin R. D., Guillou P. J., Monson J. R. (1992). Is the relationship between angiogenesis and metastasis in breast cancer real?. Surg Oncol.

[OCR_01049] Jefferson M. F., Pendleton N., Faragher E. B., Dixon G. R., Myskow M. W., Horan M. A. (1996). 'Tumour volume' as a predictor of survival after resection of non-small-cell lung cancer (NSCLC). Br J Cancer.

[OCR_01045] Kaesberg P. R., Ershler W. B. (1989). The change in tumor aggressiveness with age: lessons from experimental animals.. Semin Oncol.

[OCR_01054] Kainz C., Speiser P., Wanner C., Obermair A., Tempfer C., Sliutz G., Reinthaller A., Breitenecker G. (1995). Prognostic value of tumour microvessel density in cancer of the uterine cervix stage IB to IIB.. Anticancer Res.

[OCR_01065] Kreisle R. A., Stebler B. A., Ershler W. B. (1990). Effect of host age on tumor-associated angiogenesis in mice.. J Natl Cancer Inst.

[OCR_01069] Lauk S., Skates S., Goodman M., Suit H. D. (1989). A morphometric study of the vascularity of oral squamous cell carcinomas and its relation to outcome of radiation therapy.. Eur J Cancer Clin Oncol.

[OCR_01074] Leon S. P., Folkerth R. D., Black P. M. (1996). Microvessel density is a prognostic indicator for patients with astroglial brain tumors.. Cancer.

[OCR_01077] Macchiarini P., Fontanini G., Hardin M. J., Squartini F., Angeletti C. A. (1992). Relation of neovascularisation to metastasis of non-small-cell lung cancer.. Lancet.

[OCR_01086] Miliaras D., Kamas A., Kalekou H. (1995). Angiogenesis in invasive breast carcinoma: is it associated with parameters of prognostic significance?. Histopathology.

[OCR_01091] Morphopoulos G., Pearson M., Ryder W. D., Howell A., Harris M. (1996). Tumour angiogenesis as a prognostic marker in infiltrating lobular carcinoma of the breast.. J Pathol.

[OCR_01096] Mountain C. F. (1993). Lung cancer staging classification.. Clin Chest Med.

[OCR_01097] O'Rourke M. A., Feussner J. R., Feigl P., Laszlo J. (1987). Age trends of lung cancer stage at diagnosis. Implications for lung cancer screening in the elderly.. JAMA.

[OCR_01107] Pendleton N., Jefferson M. F., Dixon G. R., Myskow M. W., Horan M. A. (1996). Correlates of tumor size, gender, cell type, and metastasis of resected non-small cell lung cancer and age.. J Gerontol A Biol Sci Med Sci.

[OCR_01112] Révész L., Siracka E., Siracky J., Delides G., Pavlaki K. (1989). Variation of vascular density within and between tumors of the uterine cervix and its predictive value for radiotherapy.. Int J Radiat Oncol Biol Phys.

[OCR_01121] Siracká E., Révész L., Kovác R., Siracký J. (1988). Vascular density in carcinoma of the uterine cervix and its predictive value for radiotherapy.. Int J Cancer.

[OCR_01124] Tahan S. R., Stein A. L. (1995). Angiogenesis in invasive squamous cell carcinoma of the lip: tumor vascularity is not an indicator of metastatic risk.. J Cutan Pathol.

[OCR_01129] Teeter S. M., Holmes F. F., McFarlane M. J. (1987). Lung carcinoma in the elderly population. Influence of histology on the inverse relationship of stage to age.. Cancer.

[OCR_01134] Van Hoef M. E., Knox W. F., Dhesi S. S., Howell A., Schor A. M. (1993). Assessment of tumour vascularity as a prognostic factor in lymph node negative invasive breast cancer.. Eur J Cancer.

[OCR_01139] Weidner N. (1993). Tumor angiogenesis: review of current applications in tumor prognostication.. Semin Diagn Pathol.

[OCR_01147] Williams J. K., Carlson G. W., Cohen C., Derose P. B., Hunter S., Jurkiewicz M. J. (1994). Tumor angiogenesis as a prognostic factor in oral cavity tumors.. Am J Surg.

[OCR_01152] Yamazaki K., Abe S., Takekawa H., Sukoh N., Watanabe N., Ogura S., Nakajima I., Isobe H., Inoue K., Kawakami Y. (1994). Tumor angiogenesis in human lung adenocarcinoma.. Cancer.

[OCR_01157] Yuan A., Yang P. C., Yu C. J., Lee Y. C., Yao Y. T., Chen C. L., Lee L. N., Kuo S. H., Luh K. T. (1995). Tumor angiogenesis correlates with histologic type and metastasis in non-small-cell lung cancer.. Am J Respir Crit Care Med.

[OCR_01161] Yuhas J. M., Pazmino N. H., Proctor J. O., Toya R. E. (1974). A direct relationship between immune competence and the subcutaneous growth rate of a malignant murine lung tumor.. Cancer Res.

[OCR_01166] Zätterström U. K., Brun E., Willén R., Kjellén E., Wennerberg J. (1995). Tumor angiogenesis and prognosis in squamous cell carcinoma of the head and neck.. Head Neck.

[OCR_01012] de Jong J. S., van Diest P. J., Baak J. P. (1995). Heterogeneity and reproducibility of microvessel counts in breast cancer.. Lab Invest.

